# The effect of fibrin glue on the postoperative lymphatic leakage after D2-lymphadenectomy and gastrectomy in patients with gastric cancer

**DOI:** 10.1186/s12893-021-01168-5

**Published:** 2021-03-21

**Authors:** Habibollah Mahmoodzadeh, Ehsanollah Rahimi-Movaghar, Ramesh Omranipour, Mohammad Shirkhoda, Amirmohsen Jalaeefar, Seyed Rouhollah Miri, Amirsina Sharifi

**Affiliations:** 1 Department of Surgical Oncology, Cancer Institute, Tehran University of Medical Sciences, Tehran, Iran; 2 Fellowship of Oncosurgery, Cancer Institute, Tehran University of Medical Sciences, Tehran, Iran; 3 Breast Disease Research Center, Tehran university of Medical Sciences, Tehran, Iran; 4 Sina Trauma and Surgery Research Center, Tehran University of Medical Sciences, Tehran, Iran

**Keywords:** Fibrin glue, Gastric cancer, Gastrectomy, D2 dissection, Lymphatic leakage

## Abstract

**Introduction:**

Disturbance in the lymphatic drainage during D2 dissection is associated with significant morbidity. We aimed to assess the effect of fibrin glue on the reduction of postoperative lymphatic leakage.

**Methods:**

Prospective double-blinded randomized clinical trial with forty patients in each study arm was conducted. All patients diagnosed, staged, and became a candidate for D2 dissection based on NCCN 2019 guideline for gastric cancer. The intervention group received 1 cc of IFABOND® applied to the surgical bed.

**Results:**

The difference between study groups regarding age, gender, tumor stage was insignificant. (All p-values > 0.05). The median daily drainage volume was 120 ml with the first and the third interquartile being 75 and 210 ml, respectively for the intervention group. The control group had median, the first, and the third interquartile of 350, 290, and 420 ml. The difference between daily drainage volumes was statistically significant (p-value < 0.001). The length of hospital stay was significantly different between the two groups. Notably, the intervention group was discharged sooner (median of 7 Vs 9 days, p-value: 0.001).

**Conclusion:**

This study showed the possible role of fibrin glue in reducing postoperative lymphatic leakage after gastrectomy and D2 dissection.

*Registration trial number:* IRCT20200710048071N1, 2020.08.16

## Introduction

Gastric cancer is a leading cause of cancer-related mortality worldwide [1]. Historically, there was a difference between Eastern and Western countries regarding the management of gastric cancer [2]. However, both Eastern and Western guidelines emphasized the role of lymphadenectomy in local control of the disease and reported superior survival results especially in long-term follow-ups [3]. So lymphadenectomy should be performed as a part of standard procedure for resectable gastric cancers [4]. Today, the National Comprehensive Cancer Network (NCCN) advises performing regional lymphatic resection along with gastrectomy. The procedureincludes the removal of perigastric lymph nodes, named D1 dissection, and those along the vessels of the celiac axis, named D2 dissection [5].

Notably, classic D2 dissection was associated with significant morbidity including anastomotic leakage, pancreatic leakage, need to reoperation, wound infection, seroma formation, lymphocele, chylous ascites, and even systemic morbidities such as pulmonary complications [6]. Thus the classic D2 dissection which included pancreatosplenectomy has been gradually modified to involve the removal of both the greater and lesser omenta plus all the lymph nodes along the left gastric artery, common hepatic artery, celiac artery, and splenic artery [7, 8].

Oncological surgery usually involves lymph node dissection to accurately stage the tumor and to reach local control of the tumor, as well. Lymph node dissection is now considered an essential part of the surgical approach in gynecologic cancer, breast cancer, head and neck carcinomas, and melanoma [9–11]. The one common complication after lymphatic drainage disturbance in all these anatomical sites is lymphatic leakage defined as leakage of serous lymphatic fluid [12]. Regardless of the cause, lymphatic leakage causes morbidities for the patients including surgical wound dehiscence, local and systemic infection a longer stay at the hospital, risk of reoperation. Once present, the management of lymphatic leakage is a challenging issue. Therefore the best management would be prevention from development.

Recently, there has been a growing interest in the use of fibrin-containing products in these anatomical sites to prevent postoperative complications related to lymphatic drainage. Fibrin glue contains fibrinogen, factor XIII, thrombin, calcium chloride, and aprotinin. They have been utilized to secure the anastomosis of nerves, repair of dura rupture, hemostasis in cardiovascular operations, and orthopedic surgery [8]. Given the complex anatomy of perigastric lymphatic drainage, there is a high chance of disturbing normal anatomy even for experienced surgeons. According to our knowledge, there has been no study to evaluate the effect of Fibrin glue on post-D2-dissection lymphatic leakage in a randomized clinical trial. Therefore, the present study aimed to investigate the hypothesis that fibrin glue might reduce postoperative lymphatic leakage.

## Materials and methods

This prospective double-blinded randomized clinical trial was designed and constructed by the Department of Surgical Oncology, Cancer Institute, Imam Khomeini Hospital Complex, Tehran University of Medical Sciences, Tehran, Iran between August 2020 and December 2020. The ethics committee of Tehran University of Medical Sciences approved the study protocol by reference number of IR.TUMS.IKHC.REC1399.106 and was registered in the Iranian registry of clinical trials by reference number IRCT20200710048071N1 on August 16th, 2020. The clinical trial registry was based on the International Committee of Medical Journal Editors (ICMJE) guidline. All patients diagnosed with gastric cancer who were candidates to perform gastrectomy and D2 dissection based on NCCN 2019 guideline for gastric cancer have been invited to participate. Informed consent was obtained from all subjects.

Patients with the following criteria were excluded; the previous history of abdominal surgery, hematological disorders of coagulation, history of thromboembolic disease or lymphatic system disorder, allergy to aprotinin, severe pulmonary or cardiac disease, and refusal to participate in the study.

All patients underwent the same chemotherapy regimen before surgery. The chemotherapy regimens of the patients were based on NCCN’s preffered regimens and included Fluoracil leucovorin, oxaliplatin, and docetaxel (FLOT) [13]. Also, thoraco-abdominopelvic computed tomography before surgery and complete blood count, serum level of urea, creatinine, potassium, sodium, prothrombin time, partial thromboplastin time, aspartate aminotransferase, alanine aminotransferase, albumin were evaluated.

Patients were randomly assigned to the intervention group who received 1 cc of IFABOND® (Peters surgical worldwide, France) applied to the surgical bed of D2 dissection and thecontrol group who did not receive any hemostatic or placebo agent. Block randomization generated by a computer program was used to assign patients to study groups. Random block sized 4 (15 blocks) and 2 (10 blocks) were generated. The patient, the surgical attendings, and the residents who performed the follow-up sessions were blinded to the randomization sequence and therefore the study groups. Only the attending surgeon who performed the surgery became aware of randomization sequence at the conclusion of the procedure. All patients underwent midline laparotomy for gastrectomy and D2 dissection (including the removal of both the greater and lesser omenta plus all the lymph nodes along the left gastric artery, common hepatic artery, celiac artery, and splenic artery). Two 18 Fr closed drain tubes were inserted before wound closure, one of them was inserted in the surgical bed of D2 dissection and the other one was inserted in Morrison’s pouch. All the patients received the same protocol for antithrombotic and antibiotic prophylaxis adjusted for weight and comorbidities. All the procedures being carried out using ultrasonic shears (Harmonic®, Ethicon, Cincinnati, OH, USA).

Patients Data were collected on the following variables and were recorded; Baseline characteristics including age, and gender were collected preoperatively. After the procedure, pathological stage based on the 8th edition of the American Joint Committee on Cancer tumor-node-metastasis staging system for gastric cancer [14], drainage volume, and length of hospital stay.

Drain output was charted until it was less than 10 cc daily and then it was discharged. Follow-up session conducted three weeks after surgery and every three months thereafter. The sample size was calculated assuming an overall incidence of lymphatic leakage of 34.7%, based on Segural et al. [15], study. We estimated that a sample of at least 40 patients for each study arm was needed to declare an absolute 30% incidence reduction of lymphatic leakage with the treatment as significant with an alpha of 0.05 and beta of 0.80% power. All the methods used to perform this study were carried out following CONSORT 2010 guidelines [16]. Categorical variables are shown as frequency, and relative frequency and continuous variables are shown as the median. Collected data for categorical variables were compared using the chi-squared test. An independent student t-test was used to compare between the two groups at each follow-up visit and baseline. All analyses were performed by the two-sided method using Statistical Package of Social Science software (SPSS version 22; SPSS, Inc., Chicago, IL), and the p-value of < 0.05 was set as statistically significant.

## Results

The median age was 62 years old with the first and the third interquartile being 56 and 69.5 years old, respectively for the intervention group. Also, the control group had a median age of 63 years old with the first and the third interquartile of 58 and 69 years old, respectively. Participants were mostly men in each group, 31 cases (77.5%) in the intervention group and 28 cases (70%) in the control group. Tumor stage distribution in the intervention group was as follows: stage I (2 cases, 5%), stage II (10c cases, 25%), and stage III (28 cases, 70%). The Control group included 3 cases (7.5%) of stage I, 9 cases (22.5%) of stage II, and 28 cases (70%) of stage III. Mean lymph node yield was 35 ± 3 and 33 ± 2 in the intervention and control arm, respectively. (Median is presented in Table 1). There was no statistically significant difference between the two groups regarding age, gender, tumor stage, and lymph node yield (all p values > 0.05). Table 1 demonstrates the clinical characteristics of study groups.

**Table 1:** Baseline characteristics of study groups

	Intervention (n = 40)	Control (n = 40)	P-value
Age (year)	62 (56–69.5)	63 (58–69)	0.795
Gender (F/M)	9/31 (22.5%/77.5%)	12/28 (30%/70%)	0.446
Stage			0.768
I	2 (5%)	3 (7.5%)	
II	10 (25%)	9 (22.5%)	
III	28 (70%)	28 (70%)	
Lymph node yield	33 (26–41)	31 (24–46)	0.869

Data presented as median (Q1–Q3) and count (%)

The median daily drainage volume was 120 ml with the first and the third interquartile being 75 and 210 ml, respectively for the intervention group. Regarding daily drainage volume, the control group had median, the first, and the third interquartile of 350, 290, and 420 ml. The difference between daily drainage volumes was statistically significant (p-value < 0.001). The length of hospital stay was significantly different between the two groups.The intervention group was discharged sooner (median of 7 vs 9 days, p-value: 0.001). Table 2 shows daily drainage volume and length of hospital stay of study groups.

**Table 2:** Study endpoints

	Intervention (n = 40)	Control (n = 40)	P-value
Daily drainage volume (mL)	120 (75–210)	350 (290–420)	< 0.001
Length hospital stay (day)	7 (7–8)	9 (7–10)	0.001

Data presented as median (Q1–Q3)

## Discussion

In this study, we performed the first randomized clinical trial on the effect of fibrin glue on the postoperative lymphatic leakage after gastrectomy and D2 dissection. It was shown that fibrin glue could effectively reduce postoperative lymphatic leakage which leads to a reduced length of hospital stay.

The exact incidence of postoperative lymphatic drainage disturbances after gastrectomy is not clear, however, it has been reported that by increasing the extent of lymph node dissection the incidence of chyloperitoneumafter gastrectomy increases [17]. Regarding lymphorrhea, another form of lymphatic drainage disturbance, the incidencereaches 3.6% for D2 dissection plus pre-aortic lymphadenectomy [18]. Furthermore, dissection of lymph nodes along the common hepatic artery and the celiac artery increases the chance of lymphatic leakage.

Considering the significant morbidity in patients who underwent radical lymphadenectomy in different anatomical sites, different strategies have been applied to reduce the amount of lymphatic leakage which mainly can be divided into three categories. First, surgical devices for sealing vessels such as argon diathermy [19], laser scalpel, ultrasonic scalpel, and ultrasonic scissor [20], second the methods of limiting the dead space [21, 22] and third, hemostatic agents applied directly to the surgical site [9].

Primarily, fibrin glue was used in cardiovascular, liver, lung, gynecological, and urological surgery as a hemostatic agent. Moreover, upcoming studies showed their role in tissue recovery, regeneration, and faster healing [23]. Thus, it became useful in microneurosurgery [24], and gastrointestinal anastomoses [25]. Generally, the mechanism of action of different types of fibrin-containing-products is to trigger the coagulation process by activating the reaction of fibrinogen and thrombin. Upon contact with a bleeding surface, the fibrinogen-thrombin reaction transforms the active fibrinogen to fibrin and promotes the formation of the fibrin clot. Various types of fibrin-containing-products have been used worldwide and the most popular ones are Floseal®, Tachosil®, and Tissucol®. These products are different regarding the source of fibrinogen, thrombin, and aprotinin which could be of human or bovine origin. Newer products contain a human-based compound, to lessen potential immunogenic reaction [26, 27] or anaphylaxis caused by bovine aprotinin [28].

The efficacy of fibrin-containing-products has been assessed through different studies. It has been used in various types of malignancy and various anatomical sites. This divergence has led to different results about the effectiveness of these products. One of the studies in the field of evaluating the efficacy of fibrin sealant patches on the incidence of lymphatic morbidity after radial lymphadenectomy has been performed by Gasparri et al. [9]. They gathered data on 720 patients from 10 different clinical trials that used fibrin-thrombin sealant in patients who underwent axillary dissection for breast cancer, extraperitoneal dissection for prostate cancer, inguinal dissection for vulvular cancer or melanoma and pelvic dissection for endometrial cancer. The final conclusion of this study was that application of fibrin-thrombin sealant was effective in reducing postoperative lymphocele formation and reduced the need to percutaneouslly drain the seroma, the median total volume of lymph drained, and the duration of drainage. The underlying rational behind the use of these products is the fact that endothelial cells of blood and lymphatic vessels produces coagulation and fibrinolytic factors in natural hemostatic cascades and cause sealing of lymphatic capillaries, thus the use of such products may have a role in augmentation of the final stage of coagulation when fibrinogen is converted into stable fibrinogen clot [26, 29].

Gerken et al. [30] ran a systematic review and meta-analysis to investigate the preventive effect of fibrin–containing tissue sealants on lymphocele formation after radical inguinal lymph node dissection in patients with melanoma. They used six clinical trials including 194 patients. This study failed to show the effect of tissue sealants on the duration of drain placement, total drainage volume, the incidence of postoperative seroma formation, wound infection, and skin necrosis. This result might be explained as every study used a different setting regarding the surgical radicality, the size of the wound surface, the definition of drain removal criteria, and the route of application of fibrin-containing product (glue or patch). Thus, this heterogeneity in the study designs had finally caused indefinite results [31–33].

Another systematic review and meta-analysis of randomized controlled trials on the application of fibrin sealant for the prevention of lymphocele after lymphadenectomy in patients with gynecological malignancies were performed by Prodromidou et al. [28]. Four hundred eighty-one patients from six randomized clinical trials were included and it was found that fibrin sealant could significantly decrease total amount of drained fluid and mean duration of drainage. Also, there was no difference in overall incidence of lymphocele [34, 35].

The English literature about the effect of fibrin-containing products on seroma formation after lymphadenectomy is now inconclusive because there are several serious limitations in each study even systematic reviews and meta-analysis ones [36]. The lack of precise definition of seroma formation, diagnostic tools to evaluate, and appropriate prophylactic and therapeutic approaches to treat this condition resulted in a significant amount of studies without solid statement [37]. Thus to reach safe conclusion studies with a high number of included patients, unified planning and design are warranted. The present study is limited by the short followup period and limited number of participants. However, most of lymphatic dranaige disturbances occur soon after the procedure.

Finally, this study showed the possible role of fibrin glue on reducing postoperative lymphatic leakage after gastrectomy and D2 dissection, a result which should be taken cautiously because of a limited number of participants.

## Data Availability

All the data regarding study variables is available. The corresponding author will provide supplementary files up one request.
